# Immunohistochemical characterization of the immune cell response during chlamydial infection in the male and female koala (*Phascolarctos cinereus*) reproductive tract

**DOI:** 10.1177/03009858231225499

**Published:** 2024-01-19

**Authors:** Sara Pagliarani, Stephen D. Johnston, Kenneth W. Beagley, Chiara Palmieri

**Affiliations:** 1 The University of Queensland, Gatton, QLD, Australia; 2 University of Guelph, Guelph, ON, Canada; 3 Queensland University of Technology, Brisbane, QLD, Australia

**Keywords:** chlamydiosis, histology, immunohistochemistry, koala, PCR, reproductive tract

## Abstract

Chlamydiosis is one of the main causes of the progressive decline of koala populations in eastern Australia. While histologic, immunologic, and molecular studies have provided insights into the basic function of the koala immune system, the *in situ* immune cell signatures during chlamydial infection of the reproductive tract in koalas have not been investigated. Thirty-two female koalas and 47 males presented to wildlife hospitals with clinical signs suggestive of *Chlamydia* infection were euthanized with the entire reproductive tract collected for histology; immunohistochemistry (IHC) for T-cell (CD3ε, CD4, and CD8α), B-cell (CD79b), and human leukocyte antigen (HLA)-DR markers; and quantitative real-time polymerase chain reaction (rtPCR) for *Chlamydia pecorum*. T-cells, B-cells, and HLA-DR-positive cells were observed in both the lower and upper reproductive tracts of male and female koalas with a statistically significant associations between the degree of the inflammatory reaction; the number of CD3, CD4, CD79b, and HLA-DR positive cells; and the PCR load. CD4-positive cells were negatively associated with the severity of the gross lesions. The distribution of immune cells was also variable according to the location within the genital tract in both male and female koalas. These preliminary results represent a step forward towards further exploring mechanisms behind chlamydial infection immunopathogenesis, thus providing valuable information about the immune response and infectious diseases in free-ranging koalas.

*Chlamydia pecorum* infection is the most prevalent sexually transmitted bacterial disease in koalas,^20^ causing severe inflammatory lesions of the urogenital tract and infertility. While significant progress has been made in identifying vaccine targets and testing the effectiveness of different vaccines, several studies have highlighted the limited knowledge of the host immune response and the specific immune mechanisms by which the vaccines should be effective.^45,58,65,74^

It has been well documented that lesions caused by chlamydial infection are closely related to the host response to the organism, and that both the innate and adaptive host responses contribute to tissue damage.^71^ While *Chlamydia* can initiate an acute inflammatory response by eliciting cytokine and chemokine production from the host cell, the adaptive response requires activation of the cell-mediated immunity and humoral/antibody responses.

A high number of animal models have been used to describe the kinetics of the T-cell and B-cell responses to *Chlamydia* spp. in mammalian genital tissue. While there is evidence that CD4^+^ T-cells play an integral role in the resolution of *Chlamydia muridarum* and *Chlamydia trachomatis* infections in different animal models (monkey, mouse, and guinea pigs)^16,19,22^ and humans,^1,67^ the role of CD8^+^ T-cells is controversial. CD8^+^ T-cells are known to migrate to the infection site, and both human and mouse CD8^+^ T-cells have been shown to destroy cells that have been infected with *C. trachomatis in vitro*.^76^ Recent studies in mice have instead revealed that CD8^+^ T-cells are not critical for *C. trachomatis* clearance,^51,53,69^ and that the same T-cells may be implicated in *Chlamydia-*induced lesions.^43,56,72^

While the primary role of neutralizing antibodies is to reduce the initial infection burden and prevent secondary bacterial infections,^6^ there is emerging evidence supporting the idea that protein-specific *C. trachomatis* antibodies are not sufficient to protect against ascending or incidental infections.^41^ There are also studies confirming the ability of koalas to mount an effective humoral immune response against chlamydial infection; however, the role of the immune response in the infection of the genital tract has not been demonstrated in vivo yet.^13,24,34,35,37,46^

The role of the major histocompatibility complex class II (MHC-II) proteins, primarily implicated in extracellular pathogen recognition, has been extensively investigated in the koala.^30,39,40^ More specifically, preliminary studies have linked some MHC-II variants to increased resistance or susceptibility to *Chlamydia* infection.^30,38^ B-cells and MHC-II-positive cells have been studied in a limited subset urogenital tract samples of *Chlamydia*-infected koalas,^22^ but the presence of these cells has not been correlated with chlamydial load and/or the severity of lesions. In addition, the role of plasma cells as antigen-presenting cells for the induction of tissue-resident memory T-cells in the reproductive tract of female mice has been recently investigated^32^ opening up opportunities to further investigate the role of these cells in other species, including koalas.

Despite the high prevalence of the infection, there is minimal information regarding the immunopathologic response to *Chlamydia* in koalas. While histologic, immunologic, and molecular studies have provided insight into the basic function of the koala immune system,^42^ the role of the koala immune response to infectious diseases, and specifically chlamydiosis, has yet to be fully clarified. In particular, the innate and adaptive immunity stimulated by the presence of the organism in the genital tract is still unclear.^42^

Previous attempts to identify different subtypes of lymphoid cells in *Chlamydia*-infected sites have been limited to the urogenital sinus, penis, prostate, and urinary bladder.^22^ In the study conducted by Hemsley and Canfield,^22^ lymphocytes in normal and inflamed tissues consisted largely of widely distributed CD3^+^ T-cells, while B lymphocytes and plasma cells were mainly scattered beneath the epithelium.^22^ However, the unavailability of specific immunological reagents for koalas has hindered further investigations.

Histopathology and immunohistochemistry (IHC) are important tools that can assist in unraveling how the local *in situ* immune responses are responding to the pathogen, if pathological changes are present, and if chlamydial organisms can be detected. The evaluation of the *in situ* immune response in natural infections is crucial for the development of a safe and protective vaccine. Such immune responses are particularly important for diseases of the genital tract, where an exacerbated response can potentially cause infertility, as already demonstrated in female koalas.^60^ Furthermore, the formation of memory lymphocyte clusters in the reproductive tract has been suggested to be crucial for chlamydial protection,^31,32,53^ adding further support to the importance of performing a comprehensive immunohistochemical analysis of the expression of lymphoid/histiocytic markers in the genital tract of koalas. Due to the different potential roles of B-cells, T-cells, and T-cell subsets in protective immunity and immunopathology, an understanding and identification of lymphoid cells in the affected sites are thus imperative.

The present study investigated the distribution and density of different lymphocyte populations and MHC-II-positive immune cells in the entire genital tract of *Chlamydia*-positive free-ranging male and female koalas. The primary goal was to evaluate and identify potential *in situ* immune cell signatures that may correlate with protection and/or lesions during chlamydial infection in sexually mature koalas.

## Materials and Methods

### Specimens

A total of 424 tissue samples were collected from the entire genital tract of both male (*N* = 47, 188 tissue samples) and female (*N* = 32, 236 tissue samples) koalas for histologic evaluation (inflammatory score), polymerase chain reaction (PCR) analysis, and IHC for the expression of CD3ε, CD4, CD8α, CD79b, and HLA-DR. Details on the number of samples/organ are included in Supplemental Table S1. All the procedures were conducted under the approval of the University of Queensland Animal Ethics Committee (permit number SAFS/341/13/EHP/PKD and SAFS/480/16). The animals included in this study presented clinical signs suggestive of *Chlamydia* infection (eg, conjunctivitis, cystitis, cystic dilation of the ovarian bursa) and/or were positive for *Chlamydia* on real-time polymerase chain reaction (rtPCR) on conjunctival and/or urogenital swab.

### Gross Pathology Score and Clinical Scores

To correlate the severity of the clinical and pathologic lesions with the PCR load in the genital tract, only cases with a thorough description of the gross lesions, clinical information (body condition score [BCS], conjunctivitis score, rump score), and PCR performed on the entire reproductive tract were included. The gross pathology score was modified from the study of Wan et al^73^ as per Supplemental Table S2, attributing a different score to each specific organ, accounting for the severity of the lesions, and adding an additional score for the presence of fibrotic reactions in the female reproductive tract. Standardized BCS (0–10 scale),^48^ conjunctivitis (0–3 scale),^73^ and cystitis (rump) staining scores (0–3)^73^ were assigned to each female and male koala by the veterinary staff at the Currumbin Wildlife Hospital (CWH), Australia Zoo Wildlife Hospital (AZWH), and Moggill Koala Hospital as part of the koala admission procedure.

### Histopathology and Inflammatory Score

The histologic examination was performed using 5-μm-thick hematoxylin and eosin-stained sections of formalin-fixed, paraffin-embedded tissue by a blinded board-certified pathologist. The inflammatory score was recorded using specific criteria modified from the study of Paler et al^62^ and estimated based on the amount of inflammatory cells per five 40× objective fields (FN22, 1.185 mm^2^): score 0: absent (0–10 cells), score 1: mild (11–50 cells), score 2: moderate (51–150 cells), and score 3: severe (>150 cells).

### Polymerase Chain Reaction

*Chlamydia pecorum* was detected using a previously validated multiplex real-time PCR^25^ on 236 tissue samples from female koalas and 188 tissue samples from male koalas stored at −80°C. The final concentration was expressed as infectious-forming units per milliliter (IFU/ml). Based on concentration, 4 different scores of *C. pecorum* load were generated: (1) negative: 0 IFU/ml detected, (2) low PCR load: <500 IFU/ml, (3) moderate PCR load: 501 to 5000 IFU/ml, and (4) high PCR load: >5000 IFU/ml.^4^

### Immunohistochemistry

The antibodies used for IHC are listed in Supplemental Table S3. Anti-CD3ε, anti-CD4, and anti-CD8 antibodies were specifically raised against the corresponding koala proteins, while the identity between CD79b and HLA-DR human/mice and koalas and the potential cross-reactivity of the same antibodies in koala samples was initially evaluated running an NCBI (National Center for Biotechnology Information) protein BLAST (Basic Local Alignment Search Tool) and then validated by Western blot analysis using fresh koala lymphoid tissue (data not shown).

IHC was performed using an automated staining machine (Autostainer Link 48, Dako-Agilent) and integrated pre-treatment module for antigen retrieval (PT Link, Dako-Agilent). Following manual dewaxing and rehydration, the sections were incubated in the target retrieval solution at low pH (EnVision FLEX Target Retrieval Solution, Dako-Agilent) for 20 minutes at 85°C and then cooled down for 20 minutes at 65°C. Endogenous peroxidase inhibition was performed at room temperature using a pre-made solution (Dual Endogenous Enzyme Block, Dako-Agilent) for 15 minutes. A pre-diluted specific primary antibody solution (CD3ε, CD4, CD8, CD79b, and HLA-DR) was applied manually to the sections for 60 minutes at room temperature. Sections incubated with the antibody diluent (EnVision FLEX Antibody Diluent, Dako-Agilent) only and isotype controls at the same dilution rate of the primary antibodies were used as negative controls. After rinsing (EnVision FLEX Wash Buffer, Dako-Agilent), slides were incubated with the detection reagent (Dako EnVision FLEX/HRP detection reagent, Dako-Agilent). Detection was achieved using 3,3’ diaminobenzidine chromogen (EnVision FLEX Substrate Working Solution, Dako-Agilent). The sections were finally counterstained in Mayer’s hematoxylin (EnVision FLEX Hematoxylin Dako-Agilent). Well-defined membranous or cytoplasmic labeling was considered as positive expression of the antigens. Tissue samples from a koala lymph node were used as positive controls.^11,44^ A manual method was used for the IHC semi-quantitative scoring.^70^ The percentage of cells expressing positive immunolabelling out of the total population of immune/inflammatory cells was quantified and recorded as follows: score 1: 0% to 25% of positive cells, score 2: 26% to 50% of positive cells, and score 3: 51% to 100% of positive cells.

### Statistical Analysis

All the statistical analyses were performed using GraphPad Prism (GraphPad Software Inc., La Jolla, California). The Kruskal-Wallis statistical test followed by the Dunn’s multiple comparisons test was used to examine the association between selected variables. Descriptive statistics were applied to assess the percentage of inflammatory score cases out of the total cases according to the PCR load and to evaluate the mean of inflammatory scores in the 3 PCR load groups for both sexes. The significance of the difference of the inflammatory scores in the 3 PCR load groups was then confirmed by the Kruskal-Wallis statistical test followed by the Dunn’s multiple comparisons test. Correlations between PCR load, inflammatory scores, gross pathology score, all the clinical variables, and individual IHC score were determined using Spearman’s rank correlation test. The differences of the PCR load according to the sex and the gross pathology scores were evaluated using the Mann-Whitney test. A *P* value of < .05 was defined as statistically significant.

## Results

### Gross Pathology Score and Clinical Scores

Supplemental Table S4 summarizes the percentage of female and male koalas with different clinico-pathologic scores.

#### Male koalas

With respect to male koalas, 14/47 animals (29%) had information about the gross pathology scores, while BCS scores, conjunctivitis scores, and cystitis (rump) staining scores were available in 42/47 (89%), 40/47(85%), and 42/47 (89%) animals, respectively. In the 14 animals with gross lesions in the urinary and genital tract, the final gross pathology score was 1 in 8/14 (57%) and 2 in 6/14 (42%). The mean (± standard deviation) BCS was 2.8 ± 2.1 with the highest number of animals with score 2 (10/42, 23%) followed by score 5 (8/42, 19%) and 1 (8/42, 19%).

Considering the 40 animals for which the conjunctivitis score was available, grade 0 ocular disease was reported in 15/40 koalas (37%), grade 1 in 4/40 (10%), grade 2 in 6/40 (15%), and grade 3 in 15/40 (37%). The number of animals with different grades of cystitis was as follows: grade 0 in 8/42 (19%), grade 1 in 16/42 (38%), grade 2 in 5/42 (12%), and grade 3 in 13/42 (31%). The correlation matrix showed a positive correlation between BCS and conjunctivitis score (*P* = .0005) and a negative correlation between cystitis staining score and conjunctivitis score (*P* = .0002) and BCS and rump staining score (*P* = .009), while the gross pathology score was not correlated with any of the clinical variables (Supplemental Fig. S1a).

#### Female koalas

All 32 female koalas included in this study had the complete set of clinico-pathologic scores. The final gross pathology score was 1 in 16/32 animals (50%) and 2 in 16/32 (50%). The mean (± standard deviation) BCS score was 4.7 ± 1.9 standard deviation with the highest number of animals having a score of 6 (10/32, 31%), followed by scores of 4 (5/32, 16%) and 5 (5/32, 16%). Female koalas had an ocular disease grade score 0 in 13/32 animals (41%), score 1 in 8/32 (25%), score 2 in 6/32 (18%), and score 3 in 5/32 (16%). Cystitis scores were as follows: score 0 in 9/32 animals (28%), score 1 in 6/32 (19%), score 2 in 10/32 (31%), and score 3 in 7/32 (22%). The correlation matrix showed a positive correlation between the conjunctivitis score and cystitis (rump) staining (*P* = .014) and a negative correlation between the cystitis (rump) staining and BCS (*P* = .048), but no significant correlation was observed between the gross pathology score and any of the clinical variables (Supplemental Fig. S1b).

### Histopathology and Inflammatory Score

The inflammation within the reproductive tract consisted mostly of lymphocytes and plasma cells, and fewer neutrophils or, less frequently, a combination of lymphocytes, histiocytes, and neutrophils or neutrophils and lymphocytes. Mucosa-associated lymphoid aggregates were commonly described in response to local antigenic stimulation, especially in the vagina and uteri of females and in the male urethra. In males, 56/188 tissue samples (29%) had an inflammatory score of 0, 41/188 (21%) had a score of 1, 48/188 (26%) had a score of 2, and 42/188 (22%) had a score of 3. In the female reproductive tract, 115/236 samples (48%) had an inflammatory score of 0, 86/236 (36%) had a score of 1, 19/236 (8%) had a score of 2, and 16/236 (6%) had a score of 3 (Supplemental Table S5).

### PCR Load

#### Male koalas

Overall, 41% (77/188) of the tissue samples evaluated by PCR from male koalas had a low PCR load (< 500 IFU/ml), 25% (48/188) had a moderate PCR load (500–5000 IFU/ml), and 33% (62/188) had a high PCR load (> 5000 IFU/ml).

A high inflammatory score was observed in tissues with a higher PCR load (41.9% of samples with an inflammatory score of 3 had a high PCR load). Conversely, lack or mild inflammation was most commonly identified in the low PCR load group (43% of samples with an inflammatory score of 0 had a low PCR load and 26% of samples with an inflammatory score of 1 had a low PCR load). The PCR load was moderate with moderate grade of inflammation. The differences of the inflammatory scores according to the PCR load in the male genital tract was further confirmed by the Kruskal-Wallis test (*P* < .0001) and the positive correlation between inflammatory score and PCR load in the male genital tract (*r* = 0.3369; *P* < .001) (Supplemental Fig. S2).

#### Female koalas

Most of the samples from female koalas (157/236, 66%) had a low PCR load, 16% (39/236) had a moderate PCR load, and 17% (40/236) had a high PCR load. The PCR load and gross pathology scores were not significantly correlated (*r* = −0215; *P* = .2373). In the genital tract of the female koala, the absence of inflammation was observed in tissues with a low PCR load (55% of samples with an inflammatory score of 0 had a low PCR load). The differences in the inflammatory scores between the low and high PCR load were statistically significant (*P* < 0.0211).

Comparing the PCR load between males and females, there was a higher number of negative reproductive tissues in females (49% of samples were PCR-negative) compared with males (9% of samples were PCR-negative). The difference in the PCR load between males (mean PCR load = 96,129 IFU/ml) and females (mean PCR load = 22,445 IFU/ml) was statistically significant (*P* < .0001) (Supplemental Fig. S3).

### Immunohistochemistry

#### CD3ε

**Figure 1.**
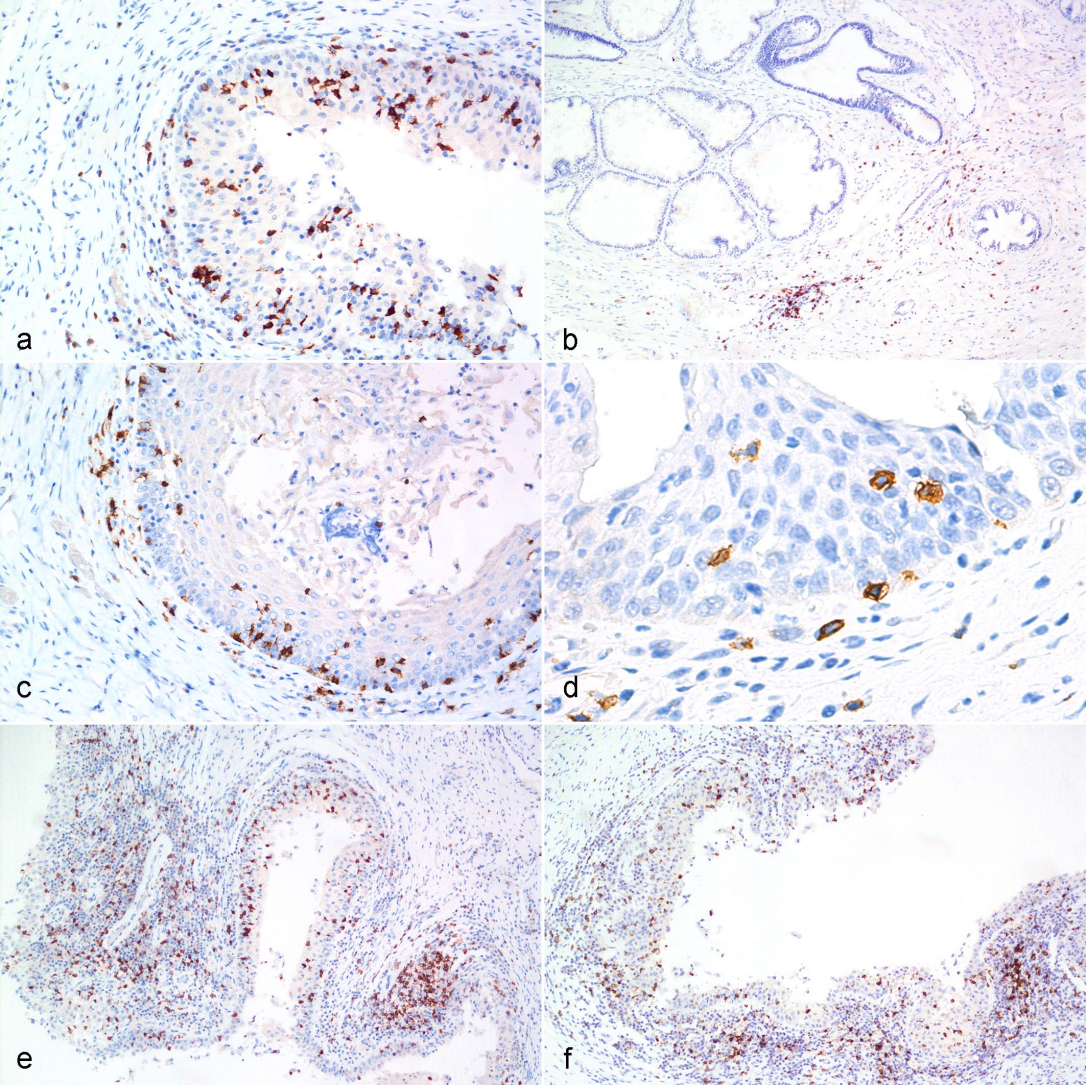
*Chlamydia pecorum* infection, reproductive tract, koala. **Figure 1a.** Presence of scattered CD3-positive cells in the epithelium and stroma of the lateral vagina. Immunohistochemistry (IHC) for CD3. **Figure 1b.** Multifocal infiltration of scattered CD3-positive cells in the stroma of the prostate. CD3 IHC. **Figure 1c.** CD4-positive cells are preferentially localized in the mucosa and superficial submucosa of the vagina with occasional transmigration within the epithelium. CD4 IHC. **Figure 1d.** Low numbers of CD4-positive cells infiltrate the epithelium of the prostatic urethra. CD4 IHC. **Figure 1e.** Randomly scattered CD8-positive lymphocytes in the mucosa and submucosa of the vagina. Note the high number of CD8-positive cells within a submucosa lymphoid nodule. CD8 IHC. **Figure 1f.** Infiltration of CD8-positive lymphocytes in the epithelium and submucosa of the prostatic urethra. CD8 IHC.

CD3ε-positive lymphocytes were distributed in the interfollicular and deep cortex of koala lymph nodes used as positive controls. In the genital organs, CD3ε-positive lymphocytes were distributed in the mucosa with occasional extension into the submucosa and transmigration in the superficial epithelial layer (Fig. 1a, b). Low number of CD3ε-positive lymphocytes was also observed in the mucosa-associated lymphoid nodules. Both in the female and male genital tracts (Supplemental Table S6), most of the tissue samples with a low PCR load had a lower number of CD3ε-positive lymphocytes (female CD3ε score 1 = 63% of tissue samples with a low PCR load; male CD3ε score 1 = 51% of tissue samples with a low PCR load). The CD3ε score differences between the low and high PCR load was statistically significant (*P* < .0001) in both males and females. PCR loads and CD3ε scores were positively correlated in females (*r* = 0.5058; *P* < .0001) and males (*r* = 0.2632; *P* = .0005).

#### CD4

CD4- positive lymphocytes were distributed in the interfollicular and deep cortex of koala lymph nodes used as positive controls. In the male and female reproductive tract, CD4-positive lymphocytes were diffusely distributed within the mucosa and superficial submucosa. Rare positive cells were also observed in the deep submucosa or transmigrating within the mucosa epithelial cells (Fig. 1c, d). In the female genital tract (Supplemental Table S6), most of the tissue samples with a low PCR load had a lower number of CD4-positive lymphocytes (CD4 score 1 = 56% of tissue samples with a low PCR load). An increased number of CD4-positive lymphocytes was observed in samples with moderate (CD4 score 3 = 43% of samples with moderate PCR loads) to high PCR loads (CD4 score 3 = 52% of samples with high PCR loads). Similarly, in the male genital tract (Supplemental Table S6), tissue samples with a low PCR load contained a lower number of CD4-positive lymphocytes (CD4 score 1 = 58% of tissue samples with low PCR loads) compared with samples with a high PCR load (CD4 score 1 = 19% of samples with high PCR loads). The differences in the CD4 score between cases with low and high PCR loads were statistically significant (*P* < .0001) in both females and males, and this was further confirmed by the positive correlation between PCR loads and CD4 scores in females (*r* = 0.2885; *P* < .0001) and males (*r* = 0.3361; *P* < .0001).

#### CD8

CD8-positive cells were located within the paracortical area of the normal koala lymph node. Within the genital tract, CD8-positive lymphocytes were diffusely distributed throughout the mucosa and submucosa layers. High numbers of CD8-positive cells populated the mucosa-associated lymphoid nodules and infiltrated the surface epithelial layer in both males and females (Fig. 1e, f). In the genital tract, most of the tissue samples with a low PCR load had a lower number of CD8-positive lymphocytes, both in females (CD8 score 1 = 54% of samples with a low PCR load) and males (CD8 score 1 = 53 % of samples with a low PCR load). The number of CD8-positive lymphocytes progressively increased in koalas with a moderate to high PCR loads. The differences in the CD8 scores between cases with low and moderate to high PCR loads were statistically significant in females (*P* < .0001) (females) and males (*P* = .01), and this was further confirmed by the positive correlations between PCR loads and CD8 scores (female: *r* = 0.3928; *P* < .0001; male: *r* = 0.2335; *P* = .0026).

#### CD79b

**Figure 2.**
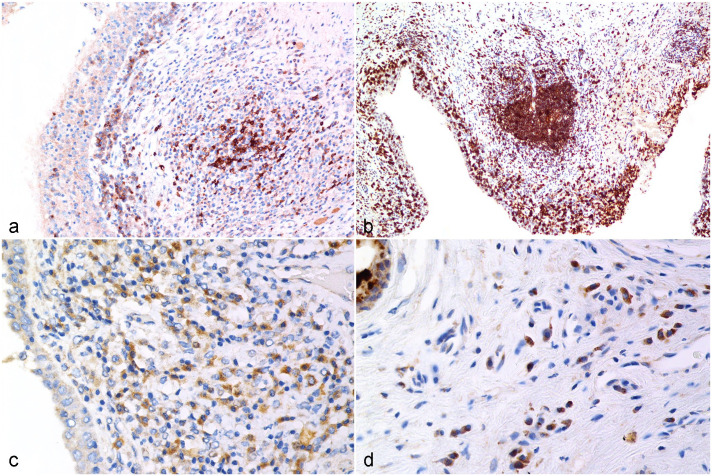
*Chlamydia pecorum* infection, reproductive tract, koala. **Figure 2a.** CD79b-positive cells are distributed in the submucosa and occasionally the mucosa of the vagina. Immunohistochemistry (IHC) for CD79b. **Figure 2b.** High numbers of CD79b-positive lymphocytes in the epithelium, submucosa, and mucosa-associated lymphoid tissue of the prostatic urethra. CD79b IHC. **Figure 2c.** HLA-DR-positive cells predominantly infiltrate the submucosa of the vagina in female koalas. HLA-DR IHC. **Figure 2d.** HLA-DR-positive cells randomly scattered within the stroma of the prostate. HLA-DR IHC.

CD79b-positive cells were observed in the follicular mantles and primary follicles in the normal koala lymph node. Strongly labeled CD79b-positive lymphocytes were observed in the mucosa and superficial submucosa of the male and female genital tracts, as well as infiltrating the surface epithelium and within the mucosa-associated lymphoid follicles (Fig. 2a, b). Tissue samples with a low PCR load contained a lower number of CD79b-positive lymphocytes in the female (CD79 score 1 = 69% of samples with a low PCR load) and male (CD79 score 1 = 58% of samples with a low PCR load) genital tract compared with the other 2 PCR load groups (Supplemental Table S6). PCR loads and CD79 scores were positively correlated in females (*r* = 0.2763; *P* < .0001) and males (*r* = 0.2108; *P* = .0062).

#### HLA-DR

HLA-DR-positive cells were mainly distributed within the follicular mantles and primary follicles of koala lymph node. HLA-DR-positive cells were mainly infiltrating the submucosa and the interstitial tissue of the male and female genital tracts (Fig. 2c, d). In the female and male genital tracts (Supplemental Table S6), tissue samples with a low PCR load had a lower number of HLA-DR-positive cells (HLA-DR score 1 = 71% of samples with a low PCR load in females and 74% of samples with a low PCR load in males) compared with moderate and high PCR loads. The HLA-DR score difference was statistically significant between the low and moderate to high PCR loads at *P* = .0136 and *P* < .0001 in the female and *P* = .0044 and *P* = .0004 in the male, respectively. PCR loads and HLA-DR scores were positively correlated in the female (*r* = 0.2813; *P* < .0001) and male (*r* = 0.3113, *P* < .0001) genital tracts.

#### Statistical analysis of immune cell populations

The Kruskal-Wallis test demonstrated statistically significant differences in the IHC scores of the different markers between the female and male genital tracts (*P* < .0001), and this was further confirmed by Dunn’s multiple comparison test. In particular, the CD4 and CD8 mean scores were significantly higher compared with the CD79b and HLA-DR mean scores in the female genital tract, while no differences were observed between the different markers in the male genital tract. However, the mean CD4, CD8, and CD3ε scores in the male genital tract was significantly higher compared with the mean CD79b and HLA-DR scores in the female genital tract.

In the female genital tract, every IHC marker (CD3ε, CD4, CD8α, CD79, and HLA-DR) showed a negative correlation with the gross pathology score. This was statistically significant for the CD4 expression (*P* = .003) (Supplemental Fig. S4).

**Figure 3.**
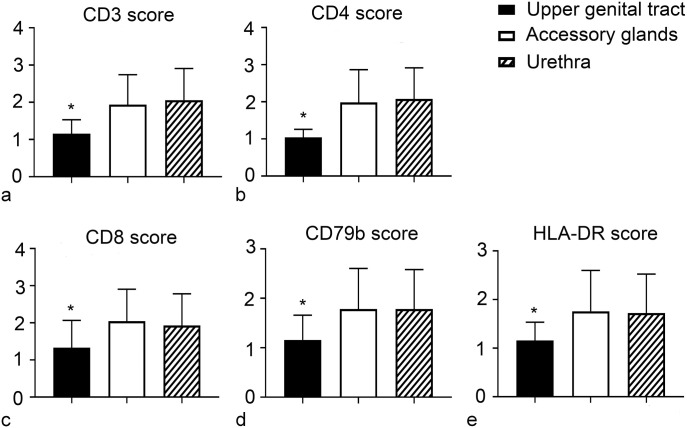
Mean immunohistochemistry scores of (a) CD3ε, (b) CD4, (c) CD8, (d) CD79b, and (e) HLA-DR in the 3 regions of the male genital system. There is a statistically significant (*) higher number of CD3ε, CD4, CD8, CD79b, and HLA-DR- positive cells in the accessory glands and urethra compared with the upper genital tract. Error bars represent the standard deviation.

With respect to the distribution of the different markers in the 3 regions of the male genital tract, there was a statistically significant higher number of CD3ε-positive cells in the accessory glands (mean score = 1.939 ± 0.80) and urethra (mean score = 2.059 ± 0.85) compared with the upper genital tract (mean score = 1.158 ± 0.37). In addition, the mean score of CD4-positive cells was higher in the urethra (2.078 ± 0.84) and accessory glands (1.985 ± 0.88) compared with the upper genital tract (1.045 ± 0.21). Similar statistically significant differences were observed between the upper genital tract and the other 2 regions in terms of CD8-positive cells, CD79b-positive cells, and HLA-DR-positive cells (Fig. 3).

**Figure 4.**
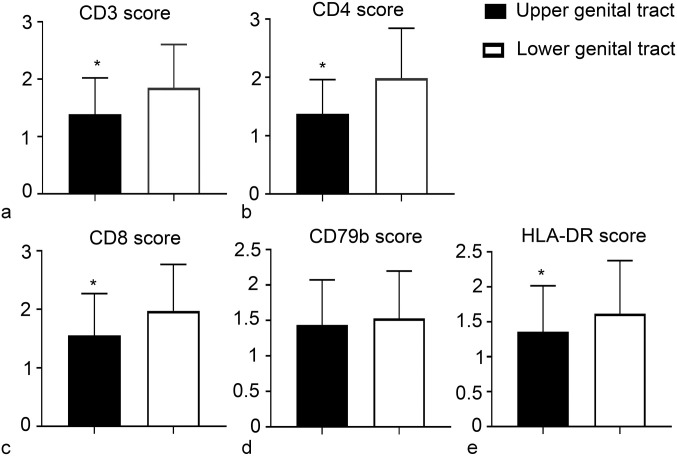
*Chlamydia pecorum* infection, reproductive tract, koala. Mean immunohistochemistry scores of (a) CD3ε, (b) CD4, (c) CD8, (d) CD79b, and (e) HLA-DR in the 2 regions of the female genital system. Statistically significant (*) higher number of CD3-positive, CD4-positive, CD8-positive, and HLA-DR-positive cells in the lower genital tract compared with the upper genital tract. Error bars represent the standard deviation.

In the female genital system, the lower genital tract showed a statistically significant higher score for CD3ε (*P* < .0001), CD4 (*P* < .0001), CD8 (*P* < .0001), and HLA-DR (*P* = .0042), while the differences were not statistically significant for the CD79b score (*P* = .2809) (Fig. 4).

## Discussion

This study has provided a quantitative assessment of T-cell, B-cell, and MHC-II-expressing cells responses in different regions of the male and female genital tract following natural infection with *C. pecorum*. Since the urogenital mucosa normally contains very few immune cells,^23,64^ a critical component of the immune clearance of *Chlamydia* is the recruitment of the appropriate lymphocyte subpopulations. The inflammatory reaction most frequently consisted of lymphocytes and plasma cells, and less frequently polymorphonuclear leukocytes. The mean inflammatory scores showed a statistically significant correlation with the 3 PCR load score groups in both male and female koalas. More inflammatory cells infiltrating the epithelial and submucosal layers of the genital tract of male and female *Chlamydia*-infected koalas were detected in the high PCR load groups (*P* < .0001). The leukocytic recruitment is most likely triggered by infected epithelial cells that can initiate an early host innate immune response^9,15,78^ to mount an appropriate immune response to the infection.

A statistically significant difference in the PCR loads between the male and female genital tracts was also reported in this present study, despite the high number of negative tissue samples detected in the female group. The presence of a lymphoplasmacytic inflammation in PCR-negative female tissue samples could be explained by the efficient recruitment of immune cells that consequently cleared the infection, since the appearance of an anti-chlamydial T-cell response in the local genital mucosa coincides with the clearance of live organisms,^10,26^ although the co-existing of other pro-inflammatory agents cannot be ruled out in natural settings. On the other side, similar to the mouse model of *Chlamydia* spp. infection,^8^ male koalas may be unable to clear the infection and the infection can persist in the upper reproductive tract, thus adversely affecting the genital environment. Nonetheless, a lower PCR load (< 500 IFU/ml) was most frequently associated with absence of inflammation in both male and female koalas, which is in line with other chlamydial studies in both human and animal models.^12,73,77^

The gross pathology scores calculated for each female and male koala based on the gross findings recorded during necropsy were not statistically correlated with the *Chlamydia* PCR loads. This is not surprising as previous studies by Palmieri et al^63^ and Pagliarani et al^61^ have demonstrated that *C. pecorum* DNA can be frequently detected by PCR in normal koala reproductive tract. Chlamydial genital infections are asymptomatic (or subclinical) in approximately 75% of women and 50% of men worldwide,^75^ and this typically contributes to a significant underestimation of the prevalence in both humans and animals.

Regarding the immunolocalization of the different types of leukocytes, our study confirmed that the koala genital *in situ* immune cell signature during chlamydial infection in the reproductive tract consists mainly of T-cells and B-cells in the sub-epithelial stroma of the lower genital tract. The immunohistochemical pattern of the lymphoid cell subpopulations was consistent with the only previously published report in koalas,^23^ and with reports in human and animal models.^2,17,50,57^ Immune cells were randomly scattered or distributed in sub-epithelial lymphoid aggregates consisting of an approximate equal number of CD3-positive, CD4-positive, and CD8-positive cells in both male and female koalas, with no statistically significant differences detected between the 2 groups. A low PCR load score was always associated with lower numbers of CD3-positive, CD4-positive, and CD8-positive lymphocytes compared with samples with high PCR load scores. However, in specific sites of the genital tract (ie, accessory glands in the male and lower reproductive tract, including the urogenital sinus, in the female), lymphocytic density did not correspond to an adequate clearance of the organism, suggesting that these sites may represent niches of *Chlamydia* proliferation and escape, favoring an ascending infection to the upper genital tract, where more severe lesions are observed, especially in female koalas. Since the local control of lymphocyte recruitment depends on a series of factors, such as the presence and concentration of chemokines and the infective dose in koalas is currently unknown, further studies examining the relationship of the levels of cytokines, chemokines, and lymphocyte recruitment would be warranted.

Another important perspective gained by evaluating the *in situ* immune response and immune cells signatures is the ability to distinguish between protective and aberrant cell-mediated responses.^60^ The vagina and cervix likely represent immune-inductive sites, with formation of mucosa-associated lymphoid aggregates in response to antigen stimulation.^66^ It has been recently hypothesized that the establishment of *C. trachomatis*-specific memory lymphocytes aggregates in the mucosa of the reproductive tract is crucial for protection against chlamydial infections.^32,35^

The presence of high number of HLA-DR-positive cells, including antigen-presenting cells and B-cells, in the genital tract is not surprising considering the ability of *Chlamydia* spp. to infect and survive within macrophages and perpetuate the infection, with *Chlamydia*-infected urethral macrophages causing testicular infection in mice.^7^ In addition to T-cells that consistently represent the predominant lymphocyte subtype in *Chlamydia* infection,^49^ B-cells were present throughout the entire reproductive tract of both male and female koalas and highly correlated with the PCR load and the presence of the organisms. The role of B-cells and antibodies in *Chlamydia* infection is quite controversial. Previous human studies have shown that *C. trachomatis*-specific antibodies play a role in chlamydial protective immunity,^3,27^ and numerous chlamydial proteins have been shown to induce antigen-specific antibodies.^18^ However, while anti-*Chlamydia* antibodies are capable of neutralizing the infection *in vitro*,^5^ increasing evidence suggests that B-cells may not be essential for combating early chlamydial infection but, instead, play a substantial role in the secondary memory response to re-infection.^52,55,71^ On the other hand, in the *C. muridarum* murine model of male genital infection, it has been recently proven that B-cells can produce interleukin (IL)-10, a potent cytokine that can facilitate pathogen survival by negatively regulating both innate and adaptive host responses.^21,68^ This might consequently delay bacterial clearance in the murine male genital tract.^68^

Another interesting finding of this study was the significant correlation between the number of CD4-positive T-cells and the gross pathology score for each organ of the entire reproductive tract of female koalas. A higher number of CD4-positive cells were associated with a lower gross pathology score. This agrees with other studies showing the pivotal role of CD4-positive cells in the protective host immune response to chlamydial infections.^28,29,33,36,54,59^

Finally, this study highlights the limitations of conducting cross-sectional studies on *Chlamydia* immune response in naturally infected koalas. While opportunistic, cross-sectional pathologic studies can be useful for research purposes, especially for an accurate qualitative description of lesions in specific target tissues, they present specific limitations, such as overrepresentation of certain demographic groups of diseased animals (ie, incurable, terminal).^14,20^ As observed in our study, the unknown, and therefore assumed, time point of infection, virulence strain of the organisms involved, disease progression, and exact reproductive status and the failure to account for all environmental stressors can significantly impact the significance of the information obtained. Despite these limitations, hospital admissions continue to be critical in acquiring valuable data at the population level in a cost-effective manner, with a high diagnostic success rate. Necropsies prove to be an effective method for the detection of subclinical and clinical *Chlamydia*-related conditions and identification of comorbidities or associated lesions.

In conclusion, the recruitment of specific T-cells, B-cells, and HLA-DR-positive cells to both the lower and upper reproductive compartments in koalas and the correlation between the CD4-positive cell number and gross pathology score in the female genital tract highlighted in this study represent a clear step forward when exploring the mechanisms behind koala chlamydial infection immunopathogenesis. This work demonstrates that, although some potential variation caused by the disease of interest are unavoidable, the evaluation of the immune cells combined with a thorough investigation of the pathologic lesions specifically in the reproductive tract, but even more generally in other affected tissues, is likely to provide meaningful information on the ability of the immune system to resolve the infection or the inability of the koala to remove the organisms and develop a non-resolving long-term infection. As species-specific reagents continue to be developed, our knowledge of immunology in koalas, and marsupials more broadly, will likely improve.^47^

## Supplemental Material

sj-pdf-1-vet-10.1177_03009858231225499 – Supplemental material for Immunohistochemical characterization of the immune cell response during chlamydial infection in the male and female koala (Phascolarctos cinereus) reproductive tract Supplemental material, sj-pdf-1-vet-10.1177_03009858231225499 for Immunohistochemical characterization of the immune cell response during chlamydial infection in the male and female koala (Phascolarctos cinereus) reproductive tract by Sara Pagliarani, Stephen D. Johnston, Kenneth W. Beagley and Chiara Palmieri in Veterinary Pathology
