# Supplementary material for: Immunohistochemical characterization of the immune cell response during chlamydial infection in the male and female koala (Phascolarctos cinereus) reproductive tract
Source: Vet Pathol. 2024 Jan 19;61(4):621–32. doi: 10.1177/03009858231225499 (PMC11264539; doi:10.1177/03009858231225499)
Supplement: sj-pdf-1-vet-10.1177_03009858231225499 – Supplemental material for Immunohistochemical characterization of the immune cell response during chlamydial infection in the male and female koala (Phascolarctos cinereus) reproductive tract [file sj-pdf-1-vet-10.1177_03009858231225499.pdf]

Supplemental Materials

Immunohistochemical characterization of the immune cell response during chlamydial infection in the male and female koala (*Phascolarctos cinereus*) reproductive tract

Sara Pagliarani, Stephen D Johnston, Kenneth W. Beagley, Chiara Palmieri

Supplemental Table S1. Number of samples collected for histopathology, PCR analysis, and immunohistochemistry for CD3ε, CD4, CD8α, CD79b, and HLA-DR markers for each section of the male and female koala reproductive tract.

	**Histology**	**PCR**	**CD3ε**	**CD4**	**CD8α**	**CD79b**	**HLA-DR**
**Male koalas**							
Upper genital tract (Total)	23	23	23	24	24	23	21
Testis	3	3	4	4	4	4	4
Epididymis	16	16	16	16	16	16	14
Vas deferens	4	4	3	4	4	3	3
Urethra (Total)	95	95	89	91	89	86	87
Prostatic urethra	37	37	36	36	35	34	34
Membranous urethra	29	29	25	29	26	28	27
Penile urethra	29	29	28	26	28	24	26
Accessory glands (Total)	70	70	67	68	69	67	68
Prostate	38	38	36	37	38	37	37
Bulbo-urethral glands	32	32	31	31	31	30	31
**Female koalas**							
Upper genital tract (Total)	80	80	59	61	61	61	61
Ovary	48	48	34	34	33	34	34
Oviduct	32	32	25	27	28	27	27
Lower genital tract (Total)	156	156	136	139	139	136	139
Uterus	37	37	34	34	33	33	34
Cervix	34	34	28	28	28	28	28
Vaginal complex[a]	63	63	53	56	57	56	57
Urogenital sinus	22	22	21	21	21	19	20

[a]Vaginal complex included lateral vaginae, median vagina, and vaginal cul-de-sac.

Supplemental Table S2. Gross pathology scoring system of the genital tract (females) and urogenital tract (males)

**Organ and gross features**		**Gross pathology score – female genital tract**
OVARY	Within normal limits	0
	Unilateral bursal cyst	1
	Bilateral bursal cyst	2
OVIDUCT	Within normal limits	0
	Hydrosalpinx	1
	Exudate or blood in the lumen	2
	Bilateral lesions	Add 0.5
UTERUS	Within normal limits	0
	Hydrometra	1
	Exudate or blood in the lumen	2
	Bilateral lesions	Add 0.5
VAGINA	Within normal limits	0
	Mild vaginitis	1
	Exudate or blood in the vagina	2
In case of fibrotic reactions alongside the reproductive tract		Add 1
FINAL SCORE	Score 1	≤ 2.5
	Score 2	> 2.5
**Organ and gross features**		**Gross pathology score – male urogenital tract**
URINARY BLADDER	Within normal limits	0
	Grade I cystitis	1
	Grade II cystitis	2
	Grade III cystitis	3
PROSTATE	Within normal limits	0
	Prostatitis	1
FINAL SCORE	Score 1	≤ 2
	Score 2	> 2

Supplemental Table S3. Primary antibodies used for immunohistochemistry.

Antibody	Type	Dilution	Source
CD3ε	mAb[a]	1:100	QUT[b] (Prof. Beagley)[*]
CD4	mAb	1:100	QUT (Prof. Beagley)[*,§]
CD8α	mAb	1:100	QUT (Prof. Beagley)[*]
CD79b	mAb	1:50 – 1:80	Santa Cruz Biotechnology (sc-53210)
HLA-DP, DQ, DR	mAb	1:200	Agilent-Dako (M0775)

[a]mAb: Monoclonal antibody

[b]QUT: Queensland University of Technology

* Madden D, Whaite A, Jones E, Belov K, Timms P, Polkinghorne A. Koala immunology and infectious diseases: How much can the koala bear? *Dev Comp Immunol*. 2018;**82**:177–185.

§ Manam S, Thomas JD, Li W, et al. Tumor Necrosis Factor (TNF) Receptor Superfamily Member 1b on CD8+ T Cells and TNF Receptor Superfamily Member 1a on Non-CD8+ T Cells Contribute Significantly to Upper Genital Tract Pathology Following Chlamydial Infection. *J Infect Dis*. 2014;**211**:1–9.

Supplemental Table S4. Summary of the percentage of female and male koalas with different clinico-pathologic scores.

Clinico-pathologic score	Males (%)	Females (%)
<u>Conjunctivitis score</u>		
Score 0	37.50%	40.60%
Score 1	10%	25%
Score 2	15%	18.70%
Score 3	37.50%	15.70%
<u>Cystitis (rump) staining score</u>		
Score 0	19.10%	28.20%
Score 1	38.10%	18.70%
Score 2	11.90%	31.30%
Score 3	30.90%	21.80%
<u>Final gross pathology score</u>		
Score 1	57.10%	50%
Score 2	42.80%	50%
<u>BCS score</u>		
Score 0	11.90%	0%
Score 1	19.05%	6.20%
Score 2	23.82%	12.50%
Score 3	7.14%	6.20%
Score 4	9.52%	15.70%
Score 5	19.05%	15.70%
Score 6	4.76%	31.20%
Score 7	2.38%	9.40%
Score 8	2.38%	3.10%
Score 9	0%	0%
Score 10	0%	0%

Supplemental Table S5. Number of samples with different inflammatory scores in each section of the male and female koala reproductive tract.

	Score 0	Score 1	Score 2	Score 3
Upper genital tract (male)				
Testis	3	0	0	0
Epididymis	16	0	0	0
Vas deferens	4	0	0	0
Urethra (male)				
Prostatic urethra	4	8	10	15
Membranous urethra	6	7	10	6
Penile urethra	3	14	10	2
Accessory glands (male)				
Prostate	7	10	11	10
Bulbo-urethral glands	13	2	8	9
Upper genital tract (female)				
Ovary	38	10	0	0
Oviduct	26	5	1	0
Lower genital tract (female)				
Uterus	9	18	3	7
Cervix	13	16	3	2
Vaginal complex[a]	27	24	9	3
Urogenital sinus	2	13	3	4

[a]Vaginal complex included lateral vaginae, median vagina, and vaginal cul-de-sac.

Supplemental Table S6. Percentage of tissue samples with different immunohistochemistry scores and PCR loads in the female and male genital tract.

		Low PCR load	Moderate PCR load	High PCR load
**CD3ε Score 1**	Male	51.39%	35.14%	26.67%
	Female	63.70%	18.42%	20.59%
**CD3ε Score 2**	Male	29.17%	35.14%	28.33%
	Female	30.14%	39.47%	58.82%
**CD3ε Score 3**	Male	19.44%	29.73%	45%
	Female	6.16%	42.11%	20.59%
**CD4 Score 1**	Male	58.11%	44.19%	19.67%
	Female	56.58%	30.77%	26.32%
**CD4 Score 2**	Male	22.97%	16.28%	32.79%
	Female	29.61%	25.64%	21.05%
**CD4 Score 3**	Male	18.92%	39.53%	47.54%
	Female	13.82%	43.59%	52.63%
**CD8 Score 1**	Male	53.52%	42.86%	29.31%
	Female	54%	17.95%	15%
**CD8 Score 2**	Male	23.94%	23.81%	25.86%
	Female	33.33%	38.46%	37.50%
**CD8 Score 3**	Male	22.54%	33.33%	44.83%
	Female	12.67%	43.59%	47.50%
**CD79b Score 1**	Male	58.46%	51.16%	40.68%
	Female	69.39%	46.15%	33.33%
**CD79b Score 2**	Male	26.15%	32.56%	27.12%
	Female	25.17%	51.28%	38.46%
**CD79b Score 3**	Male	15.38%	16.28%	32.20%
	Female	5.44%	2.56%	28.21%
**HLA-DR Score 1**	Male	73.91%	39.47%	40%
	Female	71.33%	47.37%	35.90%
**HLA-DR Score 2**	Male	14.49%	36.84%	30%
	Female	21.33%	34.21%	25.64%
**HLA-DR Score 3**	Male	11.59%	23.68%	30%
	Female	7.33%	18.42%	38.46%

Numbers are expressed as a % of cases per category (low PCR, moderate PCR, high PCR).

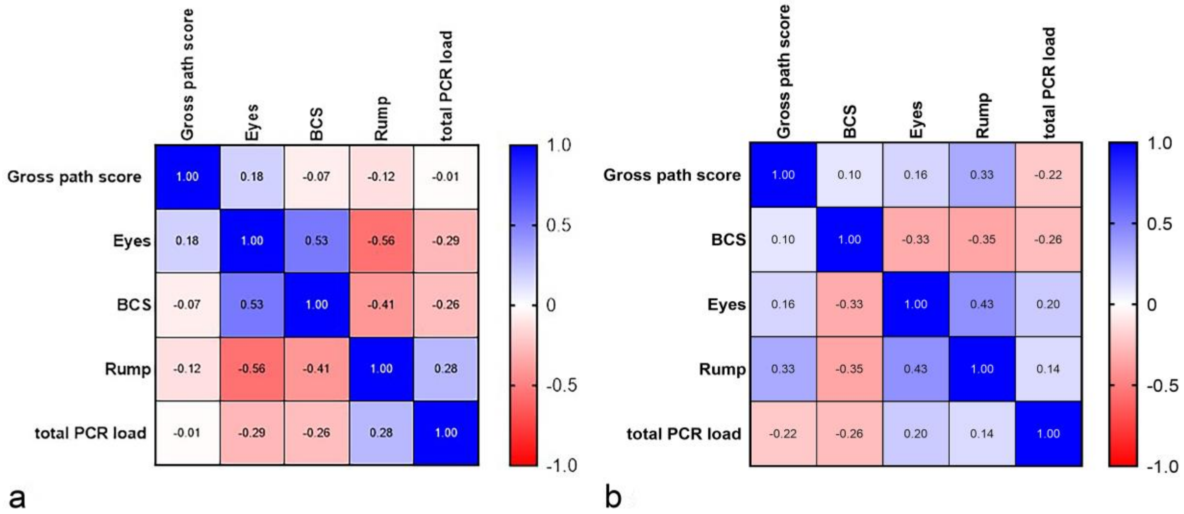

Supplemental Figure S1. Correlation matrix between the gross pathology score, conjunctivitis score (eye), body condition score (BCS), cystitis score (rump), and total PCR load in the **(a)** male and **(b)** female genital tract.

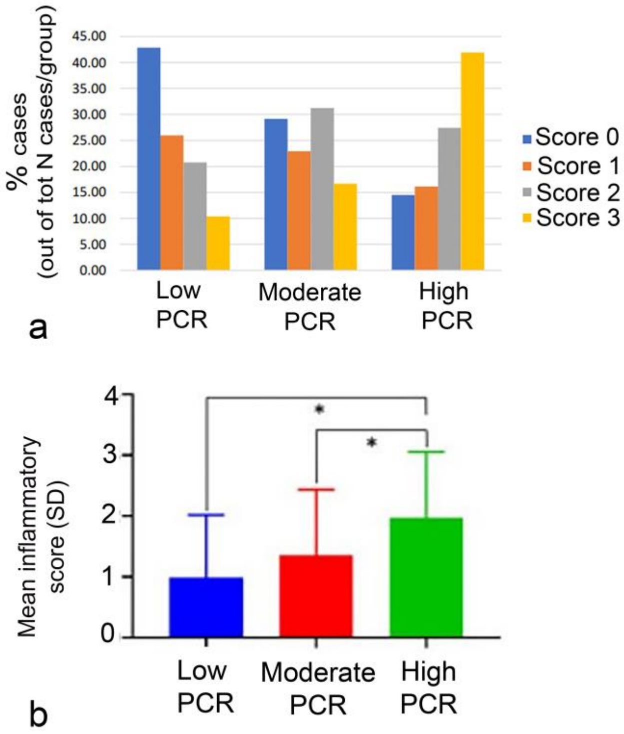

Supplemental Figure S2. (a) Distribution of the PCR load groups according to the inflammatory scores in the genital tract of male koalas. **(b)** Correlation between the inflammation score (mean) and the different PCR load group scores in the genital tract of male koalas (* statistically significant differences).

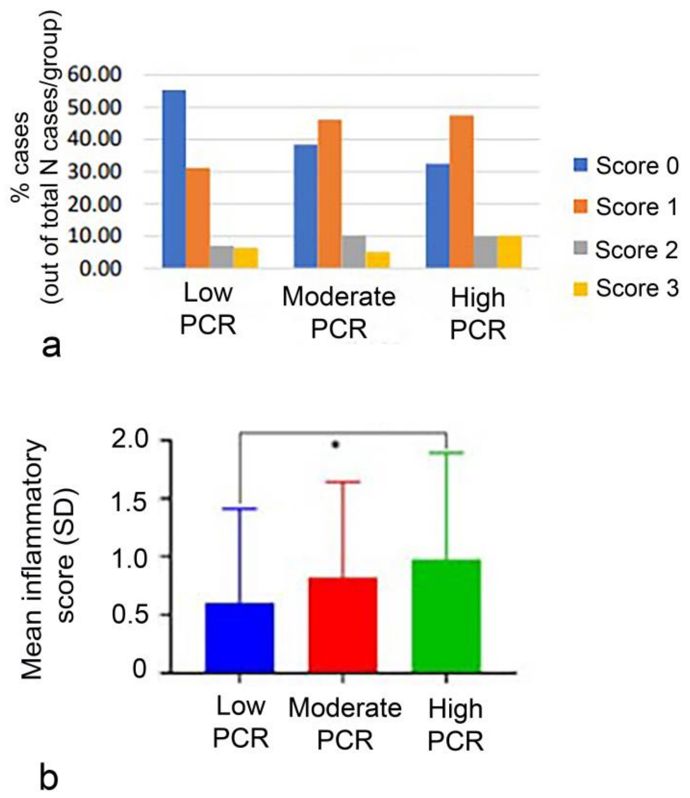

Supplemental Figure S3. (a) Distribution of the PCR load groups according to the inflammatory scores in the genital tract of female koalas. **(b)** Correlation between the inflammation score (mean) and the different PCR load group scores in the genital tract of female koalas (* statistically significant differences).

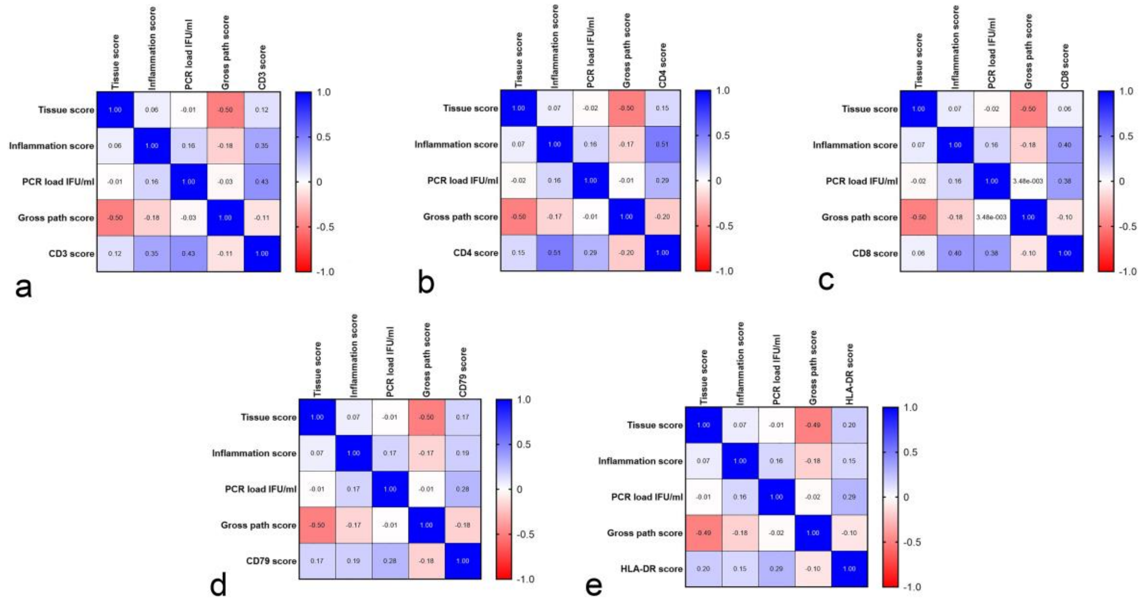

Supplemental Figure S4. Correlation matrix between the variables tissue score (type of organ), inflammatory score, PCR load, gross pathology score, and immunohistochemistry (IHC) marker scores in the female genital tract. (a) CD3, (b) CD4, (c) CD8, (d) CD79, (e) HLA-DR. All of the IHC markers showed negative correlations with the gross pathology score.
